# Total nasal reconstruction for nasal defect after treatment for extranodal natural killer/T cell lymphoma, nasal-type: A case report

**DOI:** 10.1016/j.ijscr.2019.04.033

**Published:** 2019-04-22

**Authors:** Chien M. Duong, Duy Q. Ngo, Toan D. Tran, Quy X. Ngo, Quang V. Le

**Affiliations:** a Vietnam National Cancer Hospital, 30 Cau Buou Street, Thanh Tri District, Hanoi, Viet Nam; b Hanoi Medical University, 1 Ton That Tung Street, Dong Da District, Hanoi, Viet Nam

**Keywords:** Extranodal natural killer/T-cell lymphoma, Total nasal reconstruction, Forehead flap, Rotational flap

## Abstract

**Introduction:**

Nasal-type extranodal natural killer (NK)/T-cell lymphomas are a rare type of clinical condition. Reconstruction of the complex nasal defect after chemoradiation is extremely challenging for plastic surgeons.

**Presentation of case:**

Here we present the case of a 56-year old female with the condition of a nasal-type NK/T-cell lymphoma which had caused complex nasal disfigurement. The patient had undergone chemoradiotherapy. Lesions after treatment were present all over the nasal defect (nasal septum, mucosa, support frame and skin) and the left cheek medial subunit. The surgery was subdivided into 3 stages. First, we removed the infectious tissue and restored the wall of nasal cavity by the left forehead – scalp flap. Second, we used the pedicle of the left forehead flap to rebuild the nasal mucosa defect, the rotational flap to rebuild the cheek defect, and the right forehead flap to recovered skin defect of the nose. Finally, we divided the pedicle of right forehead flap.

**Discussion:**

The complex nasal defect is difficult to reconstruct and has a higher risk of failure in patient who received chemoradiotherapy. It is crucial to choose the right materials and have a confident plan in order to achieve successful results for the sake of the patient.

**Conclusion:**

Our case report shows that the nasal defect caused by a nasal-type NK/T-cell lymphoma is complex. After our 3 stages plan for the surgery, as well as using multiple flaps for reconstruction from the forehead skin, the result was significant reduction in the disfiguration of the patient’s nose.

## Highlights

• Total nasal defect after chemoradiation was complex and difficult to rebuild.
• The surgery was divided into 3 stages with each stage to prepare for the next stage.
• With the multiple facial unit defects, reconstruction is based on aesthetic unit.
• After using multiple local flaps to reconstruct the defect, the result was improved.

The following case report has been reported in line with the SCARE criteria [1].

## 1 Introduction

Nasal-type, extranodal natural killer/T cell lymphoma (ENKL) is an extranodal lymphoma most common in Asia, as well as Central and South America, which typically causes further localized disease symptoms such nasal obstruction, epistaxis, and/or a development of destructive massed around the nose, sinuses, or palate [2]. The diagnosis of the condition is made based upon the evaluation of a specimen biopsy from the site of involvement.

Treatment of patients with nasal-type ENKL is largely determined by the extent of disease. For most patients with localized ENKL (i.e. stage I or contiguous stage II), the treatment is combined modality therapy (CMT) with concurrent radiation therapy (RT) and chemotherapy. For patients with disseminated ENKL (stage III or IV), and for those with noncontiguous stage II disease or ENKL occurring outside the upper aerodigestive tract, the use of a combination chemotherapy regimen incorporating l-asparaginase is the best treatment [3,4].

Concerning the full-thickness of the total nasal defect, in order to reconstruct, it is required to restore the inner lining, the framework and the outer covering. Using the distant free flaps is good for large, complicated lesion because it has sufficient thickness. Unfortunately, the free flap does not have a matching skin color, and we found difficulties in facial contour shaping and reconstruction in an aesthetic unit [5]. In addition, there is a high risk of failure in patients who have received chemotherapy and radiotherapy [6]. With the multiple facial unit defects, the reconstruction should based on aesthetic unit [5]. In our case, we used multiple flaps (two forehead flaps and one rotational flap) to reconstruct an aesthetic unit. The surgery was divided into 3 stages, with each stage in preparation for the next one. The advantages of our proposed method is increased safety as well as the achievement of a better functional and aesthetic results.

## 2 Presentation of case

A 56 year old female with a healthy history was admitted to the hospital 5 months after the appearance of a primary tumor of the nasal cavity that caused partial nasal obstruction. There was no evidence of the disease in other nodal or extranodal sites, such as Waldeyer's ring, gastrointestinal tract, skin, etc. Type B symptoms (fever, night sweats, weight loss) were seen in the patient at the time. Histopathological results showed that the patient has nasal-type ENKL. Furthermore, CT scans revealed a mass noted at the nasal cavity, obstructing the nasal cavity as well as disintegration of the bone structure of the nasal septum. The patient’s immune-phenotype results were CD2+, CD56, cytoplasmic CD3+, surface CD3−, CD4−, and CD8−). All the characteristics above led to the diagnosis of nasal-type ENKL, on an Ann Arbor clinical stage of IB extranodal.

The patient received a combination of modality therapy with concurrent radiation therapy (46 grays divided into 23 fractions and weekly cisplatin) and chemotherapy followed by 3 cycles of VIPD (etoposide, ifosfamide, cisplatin, dexamethasone). Evaluation post 3 months of treatment showed no signs of the tumor. However, nasal necrosis was the complication of the treatment causing full-thickness total nasal defects. As a result, the patient was transferred to the head and neck department for reconstructive surgery. The patient understood and had an adherence to the treatment process.

### 2.1 Preoperative evaluation

**Fig. 1:**
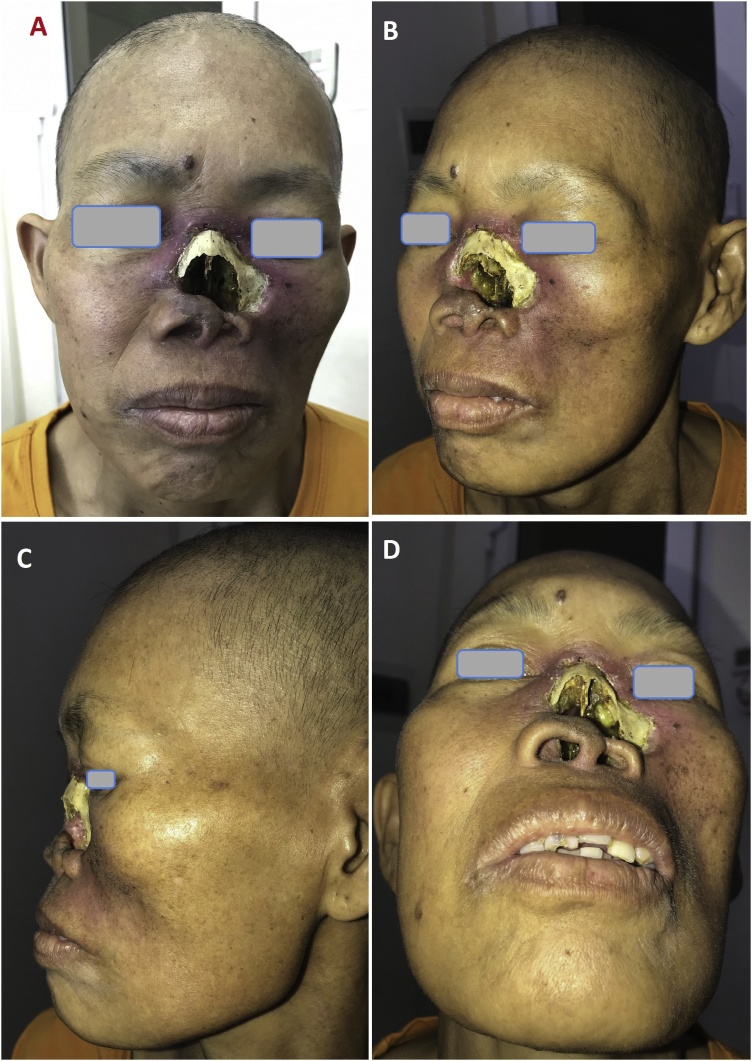
(A–D) Total nasal and cheek defect due to nasal type NK/T-cell lymphoma.

The patient’s height was 155 cm and the weight was 40 kg, with a calculated BMI = 16.6. The lesions (Fig. 1) included total nasal defect (nasal septum, mucosa of nasal cavity, support frame and skin), a part of left lower eyelid and cheek medial subunit defects. The nasal bone and frontal process of maxilla were exposed with the facial tissue being inflamed and infected, and the left cheek has atrophied due to radiotherapy.

### 2.2 Operative technique

**Fig. 2:**
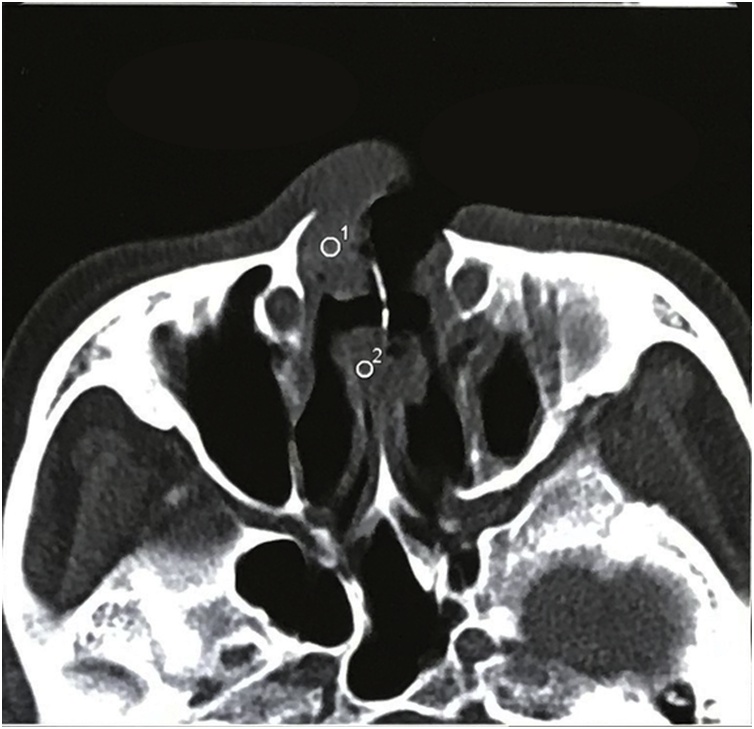
Axial computed tomographic scan showing destruction of the nasal septum, thickness of the maxillary sinus.

The surgery was subdivided into 3 “intraoperation” stages to repair the nose and cheek. It was performed by plastic surgeons (Fig. 2).

#### 2.2.1 Stage 1

**Fig. 3:**
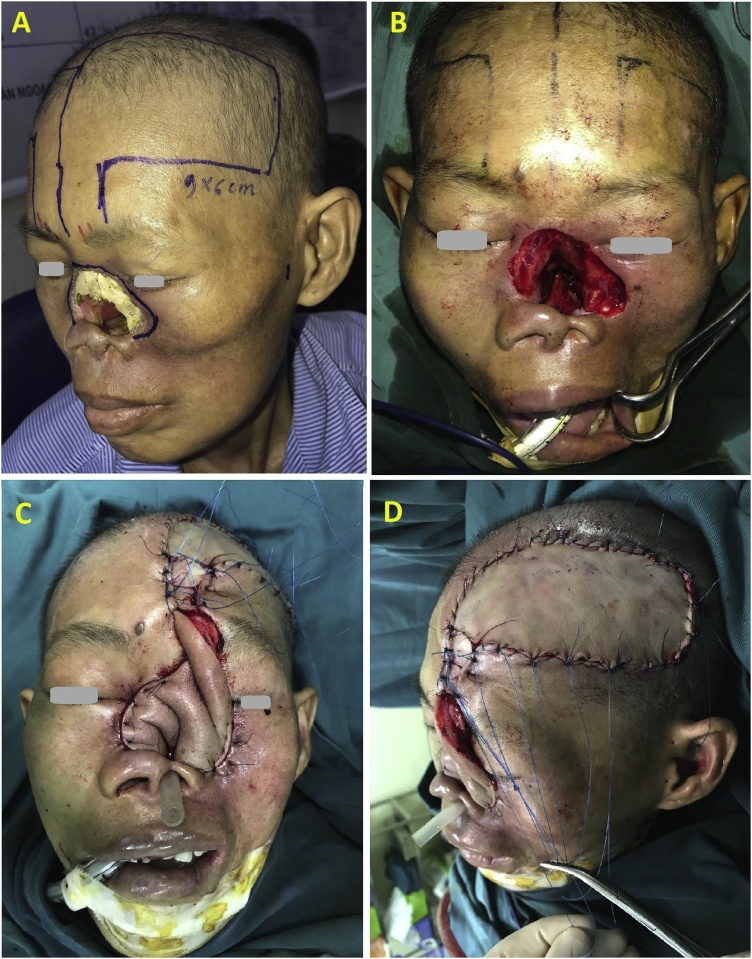
(A) Preoperation: the left forehead flap was designed, (B) First step of intraoperation: the defects included total nasal and left cheek medial subunit defects, (C) Inner lining of nasal cavity by left forehead flap, (D) Donor site required full-thickness skin graft.

The infectious tissue, necrotizing nasal and maxillary bones were removed. The lesions intraoperative include total nasal and left cheek medial subunit defects (Fig. 3B). The mucosa of the nasal cavity was repaired by left forehead – scalp flap. The size of the flap was 9 cm in width and 12 cm in length, and the one of the pedicle was 2 × 3 cm. The flap was supplied by supraorbital artery and supratrochlear artery. We used doppler handheld ultrasound to localize the arteries (Fig. 3A). The flap was harvested and moved to the nasal cavity to recover the inner lining and left cheek defect (Fig. 3C). Donor site required full-thickness skin graft (Fig. 3D).

#### 2.2.2 Stage 2

**Fig. 4:**
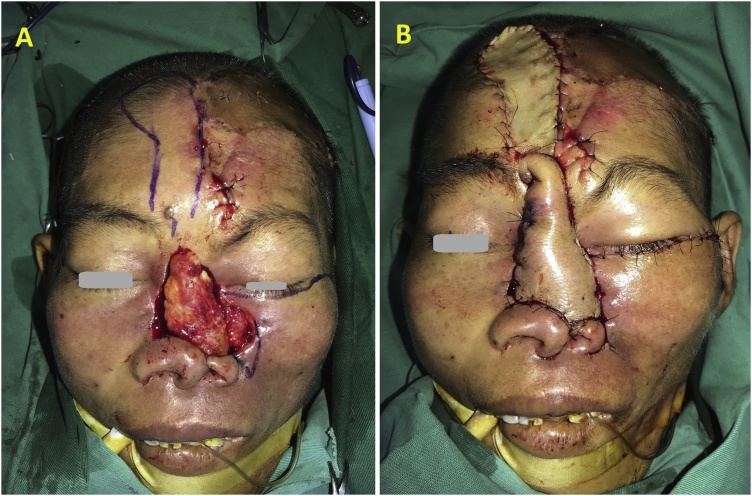
(A) The second step of intraoperation: the lesion included skin defect of the nose and left cheek. The rotational flap and the right forehead flap were designed to reconstruct. (B) Donor site of right forehead flap required full-thickness skin graft.

After three weeks from the first surgery, the flap survived completely. The pedicle of left forehead – scalp flap was divided, then turned upside down so the surface of skin was used to create the nasal wall cavity. After that the lesion included skin defect of the nose and left cheek. We used the right forehead flap, which was 4 cm in width and 6 cm in length, to recover the nasal skin defect. Donor site required full-thickness skin graft (Fig. 4B). The left rotational flap was used to recover the cheek defect (Fig. 4A).

#### 2.2.3 Stage 3

**Fig. 5:**
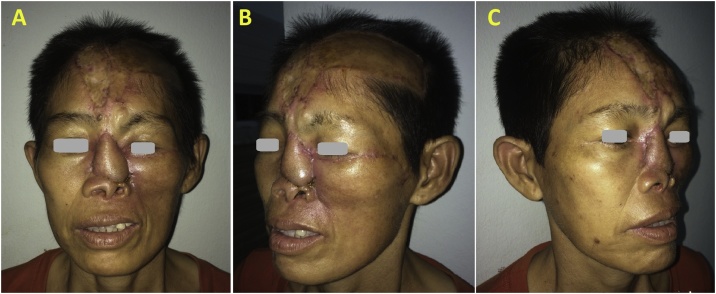
(A–C) The surgical outcome after 3 months.

Three weeks after, the pedicle of right forehead flap was divided under local anesthesia. The patient was discharged from hospital immediately after surgery (Fig. 5).

## 3 Discussion

Nasal-type ENKL is a rare clinical condition seen in only 5–8% of extranodal lymphomas of the head and neck [2]. Treatment of patients with nasal-type ENKL is largely determined by the extent of disease [3,4]. Chemotherapy and radiotherapy are the main treatments. Ionizing radiation helps to kill the tumor, however it causes damage in normal tissues located in the field of radiation. In addition, patients often lose weight due to poor nutritional status leading to weaker immune system [7]. Rarely, nasal necrosis occurs after treatment, especially in case the tumor invades to other compartments. This is an uncommon complication after chemoradiation which has not been described in literature. Thus, it is difficult for us to find the appropriate way to treat the patient’s lesion.

Severe lesions in the nose after the radiotherapy are difficult to reconstruct. Free flap can be used for total nasal reconstruction with the advantage of having sufficient thickness. However, the disadvantage is not having the color matching, difficulties in facial contour shaping and reconstruction in an aesthetic unit. In addition, there is a risk of failure in the patient who has inflamed, infectious local tissue and poor nutritional status after radiotherapy and chemotherapy.

Using multiple local flaps for total nasal reconstruction, not only ensures sufficient volume but also increases safety and can be rebuilt in an aesthetic unit principle. We used a large left forehead – scalp flap (9 × 12 cm) to line the total nasal mucosal cavity. The flap had a suitable thickness for lining the whole internal nasal cavity. At the same time, it had a big enough size to reconstruct all the walls of the nasal cavity. Some authors used preexpanded technique to increase the size of the flap without hair [[8], [9], [10]]. However, this method represents drawbacks as it lasts longer and the patient feels uncomfortable [9]. In addition, it may be susceptible to infection. The large left forehead – scalp flap had a big enough size to reconstruct all the walls of the nasal cavity. After the pedicle was divided, it was turned upside down so the surface of skin was used to create the nasal wall cavity, the subcutaneous tissue was used as recipient site for right forehead flap and the pedicle was used to rebuild dorsal nasal subunit.

The surface defects in the face should be recovered by local flap or “like” tissue is best replaced with “like” tissue, as well as the local flaps have similar characteristics to defective tissue [5]. So, we used the right forehead flap to recover the surface nasal unit defect and the left rotational flap to recover the cheek unit defect. The multiple-phasal surgery is necessary because the first, the infectious and necrotizing tissue should be removed, mucosa of the nasal cavity should be repaired. Next, supportive architecture of nose is also recipient site for the second flap should be rebuilt. Finally, the total surface nasal defect is reconstructed. After three operational phases, patient could breathe through her nose and she felt satisfied with her surgery outcomes.

## 4 Conclusions

The total nasal defect after treatment for nasal-type Nasal-type, ENKL is complex and difficult to reconstruct. After a 3 stage surgery and using multiple local flaps as material for reconstruction, the result was significantly improved function and aesthetics.

## Conflicts of interest

None.

## Sources of funding

No source to be started.

## Ethical approval

The study was approved by our research committee, Vietnam National Cancer Hospital, Hanoi, Vietnam.

## Consent

The publication of this study has been consented by the relevant patient.

## Author’s contribution

Chien M. Duong: Surgeon performed the case, wrote manuscript.

Duy Q. Ngo: Assisting surgeon operated the case, wrote manuscript.

Toan D. Tran: Assisting surgeon operated the case.

Quy X.Ngo: Follow up and post-operative management.

Quang V. Le: Surgeon performed the case.

## Registration of research studies

researchregistry4428.

## Guarantor

Quang V. Le, M.D.

## Provenance and peer review

Not commissioned, externally peer reviewed.
